# Susceptibility to Resurgent COVID-19 Outbreaks Following Vaccine Rollouts: A Modeling Study

**DOI:** 10.3390/v14102237

**Published:** 2022-10-12

**Authors:** Georgios Neofotistos, Mattia Angeli, Marios Mattheakis, Efthimios Kaxiras

**Affiliations:** 1 Harvard J. A. Paulson School of Engineering and Applied Sciences, Harvard University, Cambridge, MA 02138, USA; 2 Department of Physics, Harvard University, Cambridge, MA 02138, USA

**Keywords:** SARS-CoV-2, COVID-19 vaccines, compartmental model, vaccination coverage, waning immunity, resurgent epidemics

## Abstract

Using the recently proposed Susceptible–Asymptomatic–Infected–Vaccinated–Removed (SAIVR) model, we study the impact of key factors affecting COVID-19 vaccine rollout effectiveness and the susceptibility to resurgent epidemics. The SAIVR model expands the widely used Susceptible–Infectious–Removed (SIR) model for describing epidemics by adding compartments to include the asymptomatic infected (A) and the vaccinated (V) populations. We solve the model numerically to make predictions on the susceptibility to resurgent COVID-19 epidemics depending on initial vaccination coverage, importation loads, continuing vaccination, and more contagious SARS-CoV-2 variants, under persistent immunity and immunity waning conditions. The parameters of the model represent reported epidemiological characteristics of the SARS-CoV-2 virus such as the disease spread in countries with high levels of vaccination coverage. Our findings help explain how the combined effects of different vaccination coverage levels and waning immunity lead to distinct patterns of resurgent COVID-19 epidemics (either surges or endemic), which are observed in countries that implemented different COVID-19 health policies and achieved different vaccinated population plateaus after the vaccine rollouts in the first half of 2021.

## 1. Introduction

The World Health Organization (WHO) declared the SARS-CoV-2 outbreak in China to be a pandemic on 11 March 2020. In the two and a half years since this declaration, the disease has created unprecedented turmoil and changed the daily life of people over the entire planet, yielding a grim toll of victims that have succumbed to its attack. In the effort to contain the disease, the successful development of efficacious vaccines by late 2020, and their widespread distribution in most of the world’s countries, was hailed as the decisive and life-saving tool. Questions have lingered on whether the early COVID-19 vaccination effort will succeed in effectively containing the disease. The appearance and spread of more contagious SARS-CoV-2 strains (variants and subvariants), the onset and scale of the vaccine rollout, the high levels of vaccine hesitancy (associated with misperceptions, mistrust, low vaccine access, demographics, cost, and other factors), and the long-term persistence or lack of acquired immunity protection are among the key factors affecting the effort of disease containment through vaccination. Modeling the impact of these key factors is important for assessing the vaccination effectiveness against the pandemic.

In studying past epidemics, scientists have systematically applied “random mixing” compartmental models which assume that an infectious individual may spread the disease to any susceptible member of the population before being recovered or removed, as originally considered by Kermack and McKendrick [1]. These models comprise population compartments by considering stages of the infection and flows among the various compartments. The choice of the compartments is related to the disease that is being studied. For example, in the standard SIR model, susceptible individuals (*S*) can become infected/infectious (*I*) before being permanently recovered/removed (*R*). In the SIS model, susceptible individuals can become infected/infectious, but when they are recovered/removed, they can become susceptible again; that is, no permanent immunity is acquired. In the SEIR model, the additional compartment is the group of exposed (*E*) individuals who have become infected but are not yet infectious themselves. Recent models include a vaccinated (*V*) compartment [2,3] or compartments corresponding to many stages of infection, for instance, susceptible (*S*), infected (*I*), diagnosed (*D*), ailing (*A*), recognized (*R*), threatened (*T*), healed (*H*) and extinct (*E*), which is a model referred to as SIDARTHE [4]. Beyond compartmental models, epidemic modeling approaches involve agent-based simulations [5], heterogeneous social networks [6,7], Bayesian inference models [8], and deep learning methods [9], to name but just a few.

In the present study, we employ a compartmental model tailored to the COVID-19 epidemiological characteristics which incorporates five compartments representing the susceptible (*S*), asymptomatic infected (*A*), symptomatic infected (*I*), vaccinated (*V*), and removed/recovered (*R*) populations. We refer to this model as SAIVR. In this model, the use of the term “infected” denotes infected and infectious. Distinguishing the flows between symptomatic and asymptomatic infected populations is based on reported COVID-19 epidemiological findings [10], which indicate the average proportion of asymptomatic population to be 19% (with a 95% confidence interval of 15% to 25%) from 46 studies based on contact or outbreak investigations. In addition, as the SARS-CoV-2 virus does not appear to have the phenotypic stability of other viruses, such as those of polio or measles, infection or vaccination cannot elicit long-term protective immunity [11]. Accordingly, we have incorporated a waning immunity term in the relevant equations of the SAIVR model to control for the loss of long-term protective immunity from the SARS-CoV-2 virus.

Although a plethora of research studies are currently investigating the COVID-19 epidemiological characteristics and evolution, it appears that a simple but efficient model such as SAIVR, which can capture the basics of the complex behavior of the pandemic phenomenon including the vaccine rollout and its interdependencies, can offer useful guidance for the pandemic’s near-term and longer-term evolution. In particular, the SAIVR model is flexible enough, without being overly complicated, to allow us to extract useful information on the susceptibility to resurgent COVID-19 epidemics, and examine how this depends on initial vaccination coverage, importation loads, continuing vaccination, and more contagious SARS-CoV-2 variants, under persistent immunity and immunity waning conditions.

## 2. Materials and Methods

The original mathematical description of the spread of an infectious disease in a population is the so-called SIR model of Kermack and McKendrick [1], which divides the fixed population of *N* individuals into three compartments (also referred to as groups or classes), which evolve with time *t*:

- S(t) is the number of individuals susceptible but not yet infected with the disease;
- I(t) is the number of infected/infectious individuals;
- R(t) is the number of individuals removed (recovered) from the infected group, becoming healthy again with long-term immunity.

The SAIVR model extends the SIR model by incorporating two additional compartments representing the infected asymptomatic (*A*) and the vaccinated (*V*) populations. The model is defined by the following differential equations which determine the flows between the various compartments:

(1a) $$\begin{array}{c} \frac{dI}{dt}=\beta_{1}I\frac{S}{N}+\alpha_{2}A\frac{S}{N}+\zeta I\frac{V}{N}-\gamma I \end{array}$$

(1b) $$\begin{array}{c} \frac{dA}{dt}=\alpha_{1}A\frac{S}{N}+\beta_{2}I\frac{S}{N}+\eta A\frac{V}{N}-\gamma A \end{array}$$

(1c) $$\begin{array}{c} \frac{dS}{dt}=-\beta I\frac{S}{N}-\alpha A\frac{S}{N}-\delta \frac{S}{N}+\left(1-\lambda \right)\varepsilon V+\kappa R \end{array}$$

(1d) $$\begin{array}{c} \frac{dV}{dt}=\delta \frac{S}{N}-\eta A\frac{V}{N}-\zeta I\frac{V}{N}-\varepsilon V \end{array}$$

(1e) $$\begin{array}{c} \frac{dR}{dt}=\gamma I+\gamma A+\lambda \varepsilon V-\kappa R \end{array}$$

In the SAIVR model, the total infected population is represented by two distinct compartments, the *symptomatic* infected population (*I*) and the *asymptomatic* infected population (*A*). To ensure a constant total population (assuming no births or deaths), the following equations must be satisfied:

(2a) $$\begin{array}{c} N=S(t)+A(t)+I(t)+V(t)+R(t) \end{array}$$

(2b) $$\begin{array}{c} \alpha =\alpha_{1}+\alpha_{2},\;\;\mathrm{and}\;\;\beta =\beta_{1}+\beta_{2}. \end{array}$$

**Figure 1:**
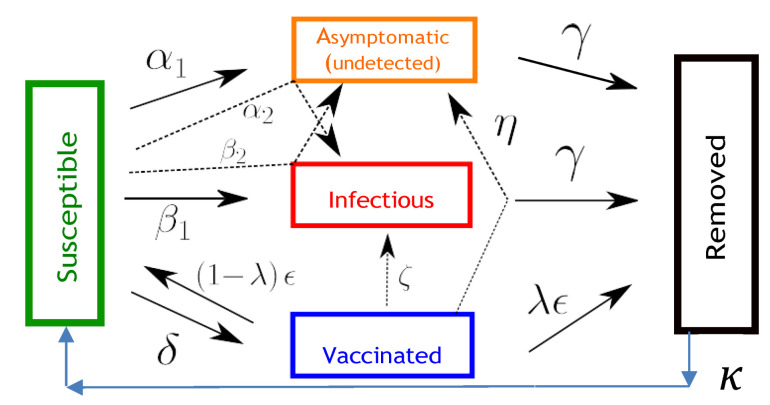
Illustration of the compartments of the SAIVR model with waning immunity (κ>0) and their interdependencies denoted by incoming and outgoing arrows and relevant flow parameters.

Figure 1 provides an illustration of the compartments of the SAIVR model with waning immunity and their interdependencies, which are denoted by incoming and outgoing arrows marked by the relevant flow parameters. The parameters of the SAIVR model have the following meaning:

- $\beta_{1}$ describes the transmission rate at which individuals are exposed to symptomatic infection, that is, an infected symptomatic individual comes into contact and infects $\beta_{1}$ other, susceptible, individuals per unit time, where the fraction that is susceptible to contracting the disease is S/N;
- $\alpha_{1}$ describes the transmission rate at which individuals are exposed to asymptomatic infection, that is, an infected asymptomatic individual comes into contact with $\alpha_{1}$ other, susceptible, individuals per unit time;
- $\beta_{2}$ describes the transmission rate at which an infected symptomatic individual comes into contact and infects susceptible individuals per unit time who become *asymptomatic* infected;
- $\alpha_{2}$ describes the transmission rate at which an infected asymptomatic individual comes into contact and infects susceptible individuals per unit time who become *symptomatic* infected;
- γ represents the mean removal (recovery) rate, that is, 1/γ is the mean period of time during which an infected individual can pass it on (is infectious) before being removed from the compartment of the infected individuals and transferred to the compartment of the removed individuals (*R* compartment);
- ζ describes the transmission rate at which a symptomatic infected individual comes into contact and infects vaccinated individuals per unit time, where the fraction of such individuals that are susceptible to contracting the disease is V/N;
- η describes the transmission rate at which an asymptomatic infected individual comes into contact and infects vaccinated, individuals per unit time;
- δ represents the vaccination rate, which is the rate at which susceptible individuals are being removed from the susceptible compartment and transferred to the vaccinated compartment; the actual number of vaccinated individuals, per day, is δ×N;
- λ represents the vaccine efficacy;
- ε represents the rate of achieving immunity after getting the vaccine dose; that is, 1/ε is the mean period of time during which a vaccinated individual has immunity before being transferred from the vaccinated compartment to another compartment, namely, susceptible with probability (1−λ), or recovered with probability λ.
- κ represents the rate at which immunity, acquired by either vaccination or infection recovery, wanes, that is, 1/κ is the mean period over which immunity is lost; upon losing immunity, the individual again becomes susceptible. We assume the same value of immunity waning for vaccinated and for infection-recovered individuals, which is an assumption corroborated by recent findings [12] showing that SARS-CoV-2 subvariants substantially escape neutralizing antibodies induced by both vaccination and infection.

## 3. Results

Our goal is to investigate, by numerically solving the equations of the SAIVR model, whether or not high levels of vaccination coverage in a community can indeed help build up a wall of protection preventing the occurrence of resurgent COVID-19 epidemics. We explore two different scenarios, one in which either infection or vaccination produce permanent immunity (κ=0), and one in which immunity is waning (κ>0). We discuss first how meaningful values of the parameters in the SAIVR model can be obtained and then expand on the importance of the waning immunity term.

Countries and states respond to the pandemic not as passive entities affected by the disease, but they actively try to protect the population by learning how to treat the infected, by adjusting the social interactions using lockdown and/or lockout measures [13], and by developing vaccines and launching vaccine rollouts. The virus also evolves, hitting populations with more contagious variants [14]. Country- or region-specific descriptions of the epidemic can be obtained by fitting the parameters of the SAIVR model to reported data. The large number of parameters involved makes their choice a challenging task. A novel fitting method based on a semi-supervised neural network [15] was applied to early 2021 data for 15 countries with high vaccination rates, and it yielded key average parameter values of $\alpha_{1}=0.20$, $\beta_{1}=0.16$, $\alpha_{2}=0.01$, $\beta_{2}=0.001$, γ=1/12, ζ=0.001, and η=0.0005, using κ=0, λ=0.95 [16], and ε=1/14, as fixed parameters.

**Figure 2:**
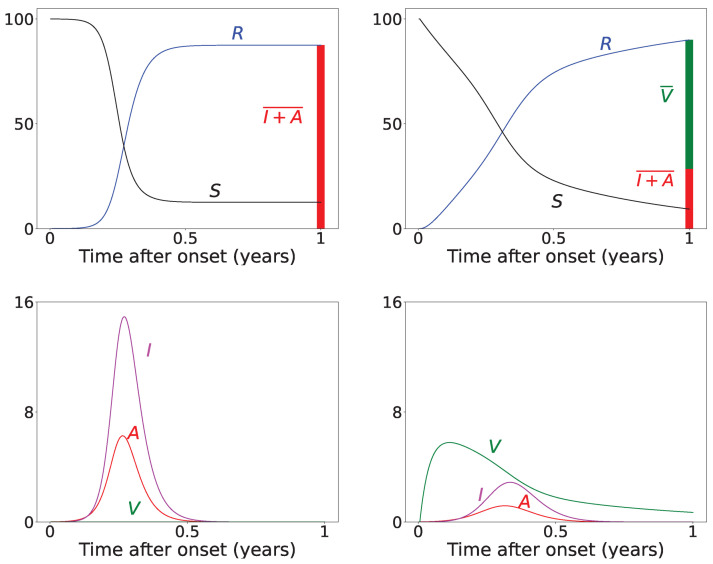
Time evolution of an outbreak obtained by numerically solving the SAIVR model, assuming persistent immunity (κ=0), acquired either by vaccination or infection recovery. The y-axis represents percentages (of the entire population, n). The x-axis represents time after the onset of the epidemic, the entire duration (*T*) of which is 1 year. **Top row:** Results without vaccine rollout (left panel) and with vaccine rollout (right panel) for the recovered population (blue curve) and the susceptible population (black curve). The red bar depicts the total number of infected individuals, $\bar{I+A}$, calculated by Equation (3); the green bar depicts the total number of effectively vaccinated individuals, $\bar{V}$, calculated by Equation (4). **Bottom row:** Symptomatic infected (*I*, magenta curve), asymptomatic infected (*A*, red curve), and vaccinated (*V*, green curve) populations, without vaccine rollout (left panel) and with vaccine rollout commencing one day after the onset of the epidemic. For both cases, $\beta_{1}=0.16$, $\alpha_{1}=0.20$, $\gamma =1/12,\beta_{2}=0.001,\alpha_{2}=0.01$, $I_{0}=10^{-4}\;N$, and $A_{0}=0.2\;I_{0}$. For the vaccination case, ζ=0.001, η=0.0005, λ=0.95, ε=1/14, and δ=0.005.

Figure 2 presents results for the time evolution of a COVID-19 epidemic, for a period of 1 year (T=1), with and without vaccine rollout, assuming permanent immunity (κ=0). For the vaccine rollout case, we have assigned δ=0.005 as a constant daily vaccination percentage of the susceptible population, which corresponds to the average daily doses administered in the USA during the first months of 2021. We also assume that the vaccine rollout commences just after the onset of the epidemic. The epidemic starts with $I_{0}=10^{-4}N$ of initially infected symptomatic individuals and $A_{0}=0.2I_{0}$ asymptomatic individuals (disease importation). The total infected population, denoted as $\bar{I+A}$, is calculated as the integral of the symptomatic and asymptomatic populations, taking into account that the infected individuals remain infected and infectious for 1/γ days before being transferred to the removed compartment:

(3) $$\bar{I+A}=\frac{1}{\gamma }\int_{0}^{T}\left(I+A\right)dt$$

As can be seen from the results of Figure 2, for these particular realizations, the vaccine rollout reduces the total infected ($\bar{I+A}$) population by about 60% compared to the epidemic evolution with no vaccine rollout. The value of the integral is depicted by the red-colored bar in the top row panels of Figure 2. In the top left panel, the removed population at the end of the epidemic stems solely from the infection-recovered individuals. In the top right panel, it stems from both the infection-recovered population ($\bar{I+A}$, red-colored bar) and the effectively vaccinated population (green-colored bar), which is denoted by $\bar{V}$ and is calculated by computing the integral of the vaccinated population, *V*, taking into account that it takes 1/ε days to obtain vaccine-induced immunity, and that only the fraction λV becomes effectively vaccinated as a result of the bounded vaccine efficacy, which is represented by the value of λ:

(4) $$\bar{V}=\frac{\lambda }{\varepsilon }\int_{0}^{T}Vdt$$

We next turn to the importance of the limited durability of immunity after vaccination or infection, which is represented by the terms that involve the parameter κ in Equation (1). This issue is the subject of ongoing investigations. Recent research findings [17] on the effectiveness of widely used vaccines (BNT162b2 of Pfizer-BioNTech, ChAdOx1 nCoV-19 of AstraZeneca, and mRNA-1273 of Moderna) in the period between 27 November 2021 and 12 January 2022 revealed that vaccine effectiveness was lower for the Omicron variant. There was no protective effect of vaccination against symptomatic disease caused by the Omicron variant from 20 to 24 weeks after the second dose, whereas effectiveness was 65.5% after 2 to 4 weeks from the second dose for those who had received two BNT162b2 doses, dropping to to 15.4% after 15 to 19 weeks. The vaccine effectiveness of two doses of the mRNA-1273 vaccine had a similar reduction over time from 75.1% after 2 to 4 weeks to 14.9% after 25 or more weeks.

In the remainder of this paper, we analyze the effect of waning immunity by making plausible assumptions for the rate of immunity loss. In each case, we assume that an initial population of recovered individuals, R(t=0) (thereafter denoted by $R_{i}$), has acquired immunity either through vaccination or infection, but this immunity will be lost over a period of time. We consider two assumptions for the range of acquired immunity, namely values of the parameter κ, as a representative range of immunity loss. We use κ=0.003 and κ=0.012, corresponding to immunity duration of 1/κ=334 days (≈11.2 months or about a year), and 1/κ=83 days (≈2.8 months or about a quarter of a year), respectively. These values provide meaningful bounds to recently reported immunity duration findings. We also produce results for κ=0.006, corresponding to 1/κ=167 days (≈5.5 months or about half a year), for comparison.

In Table 1, we present results for $R_{i}=50\%$, 70%, and 90% of the total population *N*. We note that $R_{i}$ levels in the 90% range were reported by many countries at the end of 2021 [18]: U.A.E. reported that 93% of its population was fully vaccinated, followed by Brunei, Cuba, Chile, Portugal, and the People’s Republic of China, which reported fully vaccinated percentages ranging from 89% to 85%. We study the evolution of the virus spread after the introduction of newly infected individuals in the community population, which are referred to as disease importations, denoted by $I_{0}$. We consider four cases for the value of disease importations, namely, $I_{0}=1\%$, 0.1%, 0.01%, and 0.001% of the total population *N*. The duration of the simulations is 2 years.

Table 1 presents the time evolution of the epidemics for all cases, with contact rates of $\alpha_{1}=0.20$ and $\beta_{1}=0.16$, with the remaining parameter values as mentioned in the previous section (MaterialsandMethods). The first row presents the time evolution under persistent immunity (κ=0) and no further vaccination (δ=0) of the susceptible individuals. In this case, only the 50% initial immunity protection coverage cannot prevent resurgent epidemics. With continuing vaccine rollout at δ=0.0015 (comparable to later stages of vaccine rollouts in the USA in 2021), no resurgent epidemics occur, as shown in the second row of Table 1. This fact can be attributed to the continuing vaccination, which reduces the number of susceptible individuals considerably and thus contains the virus spread. However, under waning immunity values of κ=0.003,0.006, and 0.012 (which correspond to immunity loss in about a year, half a year, and quarter of a year, respectively) and continuing vaccine rollout, with values of δ=0.0015 and 0.005, the virus spread cannot be contained, as shown in all subsequent rows of Table 1. It should be noted that higher levels of $R_{i}$ generate epidemics of higher intensities occurring at later times, as the waning immunity exposes larger segments of the population to the disease at later times. Faster waning immunity also generates more intense surges occurring at earlier times, which can be explained due to faster loss of immunity, which exposes larger segments of the population to the disease sooner. In all cases, endemic epidemics which persist indefinitely occur for all initial immunity protection levels, following the surges. Finally, as shown in the last row, under fast waning immunity (κ=0.012) and continuing vaccine rollout at a higher daily dosage (δ=0.005), resurgent epidemics are not contained but are of lower intensity and occur at a later time due to the more rigorous vaccination rollout.

It is important to note that higher importation levels generate epidemics with lower peak intensities occurring at shorter times. This issue will be discussed in detail in the next section.

## 4. Discussion

The results presented in Table 1 reveal that the concept of classical “herd immunity” may not apply to the COVID-19 epidemic as the immunity protection is not permanent, which is in agreement with recent findings [11]. Achieving high “herd immunity” levels cannot prevent surges at later times as our results indicate. This prediction of the model can explain the surge that occurred in the city of Shanghai (in the People’s Republic of China, but also in areas across Asian countries) in the spring of 2022, after almost two years of following a strict “zero-COVID” policy and very high vaccination coverage of 85.5% by the end of 2021. The surge prompted the imposition of stringent lockdowns and lockouts in Shanghai, an important industrial city and a global trade hub, which disrupted global supply chains and affected international markets.

The economic implications, social welfare, and optimal public health policy have been investigated in a recent paper by Abel and Panageas [19]. The authors use the SIR model with waning immunity and vaccination to analyze endemic steady states and disease-free states, revealing that in the long run, optimal policy leads to an endemic equilibrium (assuming reduced rates of excess deaths). Our results corroborate these findings. The average immunity protection coverage of most Western-world countries is in the range of 60–80% [18], with continuing vaccinations proceeding at a modest rate. Actual, reported, recurring surges and endemic waves continue to hit countries and states, but these surges and endemic waves show rather low intensities (flatter curves), which is in accordance with the epidemic trajectories of the model presented in Table 1, which help optimize population protection without prohibitive restrictions on the the daily lives of individuals and the economy at large.

Furthermore, our results help elucidate the effect of the size of disease importation on the temporal evolution of the disease spread. As shown in Table 1, higher numbers of importations (for instance, $I_{0}=1\%$ of the population) result in epidemics which peak faster and are of lower peak intensity (“flattened curves”). This may appear counter-intuitive but can be explained by considering that large importations increase the number of infected individuals who will soon become removed, thus keeping the removed population close to the initial level of immunity protection before waning immunity takes its toll.

To elucidate this explanation, we present in Table 2 the time evolution of the infected (I+A), removed (*R*), and susceptible (*S*) populations for four different values of disease importations ($I_{0}=1\%,0.1\%,0.01\%,0.001\%$) in a population with initial immunity protection of 50%, immunity waning rate of κ=0.006, without further vaccination (δ=0) and with continuing vaccination with δ=0.0015. For higher levels of importations, the time evolution of the virus spread is smoother; that is, the I+A curve is more flattened, although the total number of infected individuals is higher, as expected. This is rationalized by the results for the removed population, *R*, which for high levels of $I_{0}$ are not depleted as fast, and therefore, it is possible to contain disease surges by reducing the number of susceptible individuals as compared to smaller values of $I_{0}$. This finding may help explain why countries or states which have implemented “open border” policies (e.g., European Union countries, U.S. states, and countries with rather large tourist or immigration inflows) have been able to keep the impact of the contagious disease within the capacity of their healthcare systems.

The SAIVR model presented in this paper offers several advantages to help explain actual virus spread time evolution and elucidate health policy options, but it also has certain limitations. As a model of the SIR type, it compartmentalizes the population to only a few groups (S,A,I,V,R) and takes into account only importation of infected agents at t=0. We note that importations at later times can be easily incorporated in the model, resulting in, essentially, a superposition of epidemics. More contagious variants can also be modeled (with larger values of the α and β coefficients), giving qualitatively similar behavior but with larger peak values occurring in shorter time scales. The emergence of new variants and subvariants may lead to different characteristics whose epidemiological parameters are not known for some time after the variant has been identified. This presents considerable challenges for the models, which rely on accurate parameter estimates to make useful predictions for health policies implementation.

SAIVR is a simple deterministic model, which does not take into consideration age, gender, spatial distribution, births or deaths, and any other factors that may be relevant for a more realistic description of the epidemic. It assumes homogeneous mixing; that is, individuals make contact at random, and the transmission, recovery and vaccination rates are the same for all individuals. In addition, the total population size is constant and large. Its main strength is its simplicity and the insight it offers on how key epidemiological variables (including vaccination variables and waning immunity) affect populations in countries, states, or cities. The results derived from this model can be useful for a quantitative assessment of the vaccine rollout characteristics to contain the pandemic.

## Figures and Tables

**Table 1:** Time evolution of infected (I+A) following disease importations.

1/κ (Days)	δ	$R_{i}=50\%$ (of *N*)	$R_{i}=70\%$ (of *N*)	$R_{i}=90\%$ (of *N*)
*∞*	0	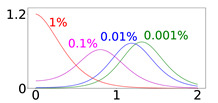	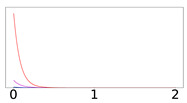	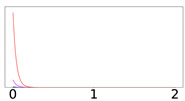
*∞*	0.0015	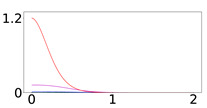	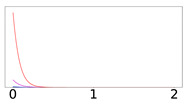	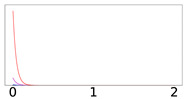
334	0.0015	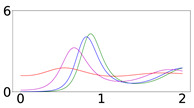	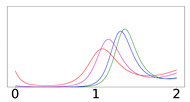	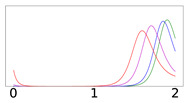
167	0.0015	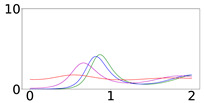	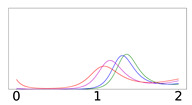	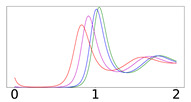
83	0.0015	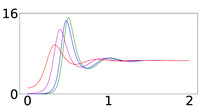	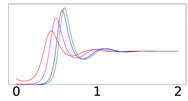	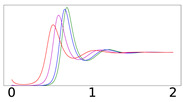
83	0.005	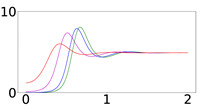	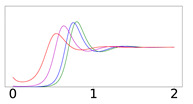	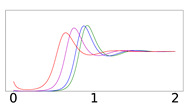

Time evolution of infected (*I* + *A*) populations following the introduction of disease importations in a population with initial immunity protection *R_i_* at 50% (left panels), 70% (middle panels), and 90% (right panels) of the total population *N*, for four different importations, *I*_0_ = 1% (red curve), 0.1% (magenta curve), 0.01% (blue curve), and 0.001% (green curve). Results are obtained for *β*_1_ = 0.16 and *α*_1_ = 0.2 (see text for details and for the values of the other parameters), for a time period of 2 years (x-axis). The y-axis scale is not the same for all plots, but it is the same in each row; for all plots, it represents percentage of the total population. Different rows correspond to different assumptions for immunity, ranging from persistent immunity (*κ* = 0 or 1 /*κ* = ∞) to waning immunity over a period of about a quarter of a year (*κ* = 0.012 or 1 /*κ* = 83 days). Vaccination dosages also vary from no vaccination (*δ* = 0) to rollouts that correspond to *δ* = 0.0015 and *δ* = 0.005

**Table 2:** Time evolution of infected (I+A), removed (*R*), and susceptible (*S*) populations.

No Vaccination	Continuing Vaccination
(δ=0)	(δ=0.0015)
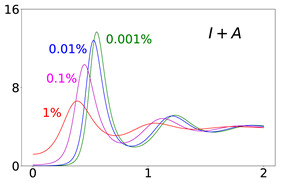	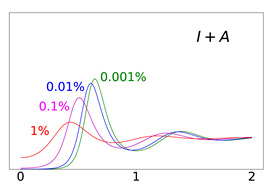
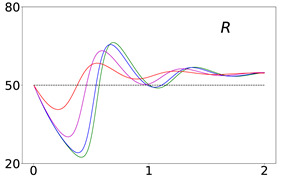	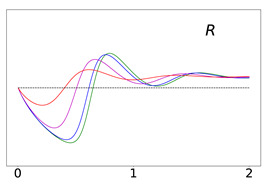
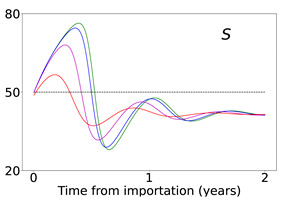	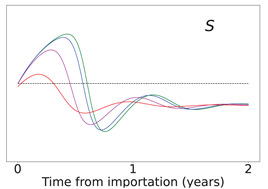

Time evolution of the infected (*I* + *A*, top row), removed (*R*, middle row), and susceptible (*S*, bottom row) populations, with initial immunity protection at 50%, for four different disease importations, *I*_0_ = 1% (red curve), 0.1% (magenta curve), 0.01% (blue curve), and 0.001% (green curve) of the total population *N*. Results are obtained for *β*_1_ = 0.16, *α*_1_ = 0.20 with waning immunity *κ* = 0.006, for the case of no further vaccination (*δ* = 0, left column) and continuing vaccination (*δ* = 0.0015, right column). The scales in the y-axis represent percentages of the total population. The black dashed horizontal line represents the 50% initial immunity level.

## Data Availability

The data presented in this study are available on request from the corresponding author.
